# Evaluation of the effect of a long inspiratory ramp time during pressure-controlled ventilation with volume guarantee: study protocol for a randomized, controlled trial

**DOI:** 10.1186/s13063-026-09888-w

**Published:** 2026-07-07

**Authors:** Johannes Hell, Jonas Manke, Stefan Schumann

**Affiliations:** 1 Department of Anesthesiology and Critical Care, University Medical Center Freiburg, Freiburg, Germany; 2 Faculty of Medicine, University of Freiburg, Freiburg, Germany

**Keywords:** Mechanical power, Lung-protective ventilation, Compliance, Inspiratory ramp time, Postoperative pulmonary complications

## Abstract

**Background:**

The inspiration of pressure-controlled ventilation is characterized by an initial sharp increase of flow rate. The resulting peak flow rate could induce a disproportionately high mechanical load, transferred from the ventilator to the pulmonary structures. A higher inspiratory ramp time could decrease the peak inspiratory flow rate, which might reduce the mechanical power transmitted to the lungs. This study compares pressure-controlled ventilation with volume guarantee with low and high inspiratory ramp time and the effects on the mechanical load transferred from the ventilator to the patient’s lungs.

**Methods:**

This is a randomized, controlled single-center trial, in which patients receive pressure-controlled ventilation with volume guarantee with short (clinical standard) or increased inspiratory ramp time. Mechanical power will be compared between the two types of ventilation. Further, the effects of the respective ventilation setting will be evaluated with the Horowitz index, the mean airway pressure, and changes in respiratory system compliance. The occurrence of atelectasis will be investigated utilizing electrical impedance tomography. Additionally, lung-specific proteins in patients’ blood will be measured, seeking indications of mechanical stress in their dynamics and postoperative pulmonary complications will be recorded and compared between groups.

**Discussion:**

This trial aims to evaluate the feasibility of decreasing inspiratory flow rate during pressure-controlled ventilation with volume guarantee by increasing inspiratory ramp time and to analyze its effects on mechanical power, oxygenation, respiratory system mechanics, atelectasis, and postoperative pulmonary complications. Furthermore, lung-specific proteins in patients’ blood will be examined in regard to find a novel, helpful biomarker, representing the mechanical load of the lungs. The results of this study could provide valuable information to improve perioperative mechanical ventilation with a reduced mechanical power through a simple an ubiquitously available modification of the breathing pattern, namely increasing inspiratory ramp time. Additionally, identification of a lung-specific biomarker reflecting mechanical load of the lungs might enhance research of lung-protective ventilation.

**Trial registration:**

German Clinical Trials Register (DRKS), DRKS00035496. Registered on 12 November 2024; URL: https://drks.de/search/en/trial/DRKS00035496/details.

Local Trials Register: Freiburger Register of Clinical Studies (FRKS), FRKS005304.

## Introduction

### Background and rationale {6a}

The concept of lung-protective ventilation has been the subject of research for many years. Since the establishment of positive end-expiratory pressure, and the concepts of low tidal volume, respectively low peak inspiratory pressure (driving pressure), current concepts of lung-protective ventilation focus on mechanical power and its reduction [1]. As part of this approach, respiratory gas flow is mentioned in many studies as another possible parameter that could influence ventilator-induced lung injury [1–8]. Reduction of the gas flow could decrease wall shear stress in the conducting airways which mainly depends on airflow velocity and smoother alveolar stretching through a decelerated time profile of the breathing pattern.

In own unpublished preliminary physical simulations with computational fluid mechanics, a reduction of peak inspiratory flow from 60 l∙ml^−1^ to 40 l∙ml^−1^ reduced the velocity of air by 32% with a consecutive 46% reduction of wall shear stress. In a cell model, smoother elongation of bronchus epithelial cells has been associated with reduced production of inflammatory cytokines and oxidative stress [9]. Moreover, limitation of the expiratory gas flow reduced lung damage in animal models [10–13]. Regarding the inspiration, there is only an animal study suggesting that a lower peak inspiratory flow could reduce lung damage [14]. However, the extent to which these results can be transferred to humans is unclear.

During pressure-controlled ventilation with volume guarantee, a lower inspiratory flow rate can be easily achieved by increasing the inspiratory ramp time. In an unpublished preliminary investigation in a physical model of the respiratory system, receiving pressure-controlled ventilation with volume guarantee, a higher inspiratory ramp time resulted in reduced mechanical power compared to a lower inspiratory ramp time.

Therefore, we designed this study to compare perioperative pressure-controlled ventilation with volume guarantee with high to low inspiratory ramp time. We hypothesized that patients ventilated with an increased inspiratory ramp time would be loaded with less mechanical power. To assess the potential relationship between the physical variable and biological effects and with the intention to identify a biomarker, which may indicate the mechanical load of the lungs, we evaluate lung-specific proteins and their kinetics during mechanical ventilation.

### Objectives {7}

The primary aim of this study is to compare mechanical power of patients ventilated with pressure-controlled ventilation with volume guarantee with an increased vs. clinical standard short inspiratory ramp time. Secondary objectives are the evaluation of ventilation efficiency between the two groups in terms of oxygenation, respiratory system mechanics, emergence of atelectasis, and postoperative pulmonary complications. Furthermore, we evaluate the kinetics of lung-specific proteins in patients’ blood in the course of mechanical ventilation.

### Trial design {8}

The study design is reported in accordance with the SPIRIT 2013 Checklist. This is an exploratory pilot study that is monocentric, prospective, randomized, and controlled with blinded patients, unblinded data collectors and blinded evaluators. The study is conducted at the Department of Anesthesiology and Critical Care, Medical Center – University of Freiburg and has been approved by the local Ethics Committee of the University of Freiburg (24-1273_S1/24-1273_1-S1). The trial complies with the principles of the Declaration of Helsinki and the principles of Good Clinical Practice.

## Methods: participants, interventions, and outcomes

### Study setting {9}

The study will be conducted at the Department of Anesthesiology and Critical Care at the Medical Center - University of Freiburg and the Department of Neurosurgery at the Medical Center - University of Freiburg. All patients will undergo neurosurgical procedures to ensure a longer time of mechanical ventilation with minor tissue damage.

## Eligibility criteria {10}

Inclusion criteria:

1. Patients scheduled for elective brain surgery with an expected duration of at least 4 h in supine position
2. Patients must be able to give informed consent

Exclusion criteria:

1. Patients under the age of 18
2. Patients are unable to give consent
3. Body mass index greater than 35 kg/m^2^
4. American Society of Anesthesiologists physical status classification >3
5. Patients with a pacemaker, implantable cardioverter-defibrillator, or cardiac resynchronization therapy
6. Pregnancy
7. Anemia
8. Patients with terminal pulmonary disease
9. Long-term use of immunosuppressants

Drop-out criteria:

1. Transfusion of blood products

### Who will take informed consent? {26a}

The principal investigator will obtain written informed consent from potential participants. After patient screening, potential participants will receive information about the study on the day before their surgery. This information will include both spoken and written details about the study, its procedures, and any risks. Patients can refuse their consent at any time without explanation.

### Additional consent provisions for collection and use of participant data and biological specimens {26b}

There are no additional consent provisions for collection and use of participant data and biological specimens in ancillary studies.

## Interventions

### Explanation for the choice of comparators {6b}

The aim of this study is to investigate the mechanical load transferred to patient’s lungs in dependence of the inspiratory ramp time during pressure-controlled ventilation with volume guarantee. Mechanical power is the most recent variable for assessing the delivered mechanical energy to the patient’s respiratory system [1]. Furthermore, the mechanical power is sensitive enough to discriminate between fine adjustments of mechanical ventilation to assess their potential contribution to improved lung-protective ventilation. Additionally, mechanical power can be determined reliably and continuously over a longer period of ventilation without additional risks for the patients.

## Intervention description {11a}

### Ventilation protocol

In this study, patients will receive a pressure-controlled ventilation with volume guarantee with a tidal volume of 8 ml∙kg^−1^ of predicted body weight, with an inspiratory oxygen fraction between 0.3 and 0.5. The breathing rate will be adjusted to maintain end-expiratory carbon dioxide partial pressure in the range of 32 to 45 mmHg. Positive end-expiratory pressure will be set at 7 cmH_2_O for all patients. Patients of the intervention group will be ventilated with the longest available inspiratory ramp time permitted by the ventilator and with an inspiratory-to-expiratory time ratio of 1:1 to 1:1.2 to facilitate the longest possible inspiratory ramp time. Patients of the control group will be ventilated with a short inspiratory ramp time (0 s) with an inspiratory-to-expiratory time ratio of 1:1.9 to 1:2.1.

### Anesthesia

Besides ventilation settings, both patient groups will receive the same standardized anesthetic management. After establishing standard monitoring (non-invasive blood pressure measurement, pulse oximetry, electrocardiogram), and additional placement of an electrode belt for electrical impedance tomography (EIT), patients will be preoxygenated with a tight-fitting face mask to achieve an end expiratory oxygen fraction > 0.8. Subsequently, anesthesia induction with a bolus of Sufentanil (20–50 µg) and a target-controlled infusion of propofol (5.0 to 7.0 μg·ml^−1^ effect site concentration, Schnider model) will be started, followed by relaxation with rocuronium (0.6 mg·kg^−1^ body weight). Depth of anesthesia will be measured with the BIS VISTA Monitoring System (Covidien, Dublin, Ireland). Three minutes after administration of the relaxant, endotracheal intubation will be performed. Afterwards, ventilation according to the protocol will be started until the end of the surgical procedure. An arterial line will be established for continuous blood pressure measurement and arterial blood gas analysis. Perioperative anesthesia will be maintained with a target-controlled infusion of propofol and a continuous infusion of remifentanil 0.2–0.4 μg∙kg^−1^∙min^−1^ aiming for a bispectral index between 20 and 50. A combination with inhalation of 0.3–0.5 MAC sevoflurane may be used if required. Fluid management will be performed in standardized fashion according to the institutional protocol.

### Criteria for discontinuing or modifying allocated interventions {11b}

The attending anesthesiologist can intervene during the complete period of ventilation, if, in his opinion, the modification of ventilation poses a risk to the patient. The anesthesiologist may modify or discontinue the assigned mode of ventilation. At this point, further recording of ventilation data will be stopped and only data up to this point will be included in the evaluation. Furthermore, in case of an increased blood loss, requiring blood transfusion, collecting blood for the study will stop and the patient will be excluded from further participation in the study.

### Strategies to improve adherence to interventions {11c}

The study director will oversee data collection and conduct regular checks to ensure adherence to intervention protocols. If there are any mistakes in recorded data, this will be reported to the study director, who will take further action.

### Relevant concomitant care permitted or prohibited during the trial {11d}

Both study groups receive comparable concomitant care and will be treated equally.

### Provisions for post-trial care {30}

After the surgical intervention, the patients will be transferred either to the post anesthesia care unit and then to the regular ward or to the intensive care unit, based on their specific circumstances. All patients will be monitored by the study team for 7 days after surgery to detect any postoperative pulmonary complications or other adverse events. If postinterventional complications occur, they will be reported to the study director, who will take further action.

### Outcomes {12}

The study compares two strategies of ventilation, focusing on the effects of a long compared to a short inspiratory ramp time during pressure-controlled ventilation with volume guarantee.

### Primary endpoint

- Mean mechanical power. Mechanical power, and mechanical power corrected for endotracheal tube resistance, will be calculated from the continuously recorded inspiratory volume-pressure loop during mechanical ventilation, and compared between groups.

### Secondary outcome measures

- Horovitz index. Horovitz index will be calculated as the quotient of blood carbon dioxide partial pressure and inspiratory oxygen fraction hourly during mechanical ventilation and on the morning of the first postoperative day if available.
- Measures of respiratory system mechanics such as mean airway pressure or changes in respiratory system compliance (ΔC) in the course of mechanical ventilation. Mean airway pressure and the change in respiratory system compliance, relative to baseline compliance at introduction, will be compared between groups.
- Change of lung-specific protein concentrations depending on duration of ventilation and study groups. Blood samples will be taken after anesthesia induction, at the end of surgery and on the morning of the first postoperative day to measure concentration of lung-specific proteins.
- Change of inflammatory proteins. After anesthesia induction, at the end of surgery and on the morning of the first postoperative day, a blood sample will be taken to measure inflammatory markers.
- Changes of the electrical impedance of the lung during ventilation. During anesthesia induction and hourly during surgery, EIT series will be recorded to evaluate lung aeration.
- Frequency of postoperative pulmonary complications. During the 7 days following surgery, patients will be screened for postoperative pulmonary complications according to a standardized protocol.

### Participant timeline {13} (Fig. 1)

**Fig. 1:**
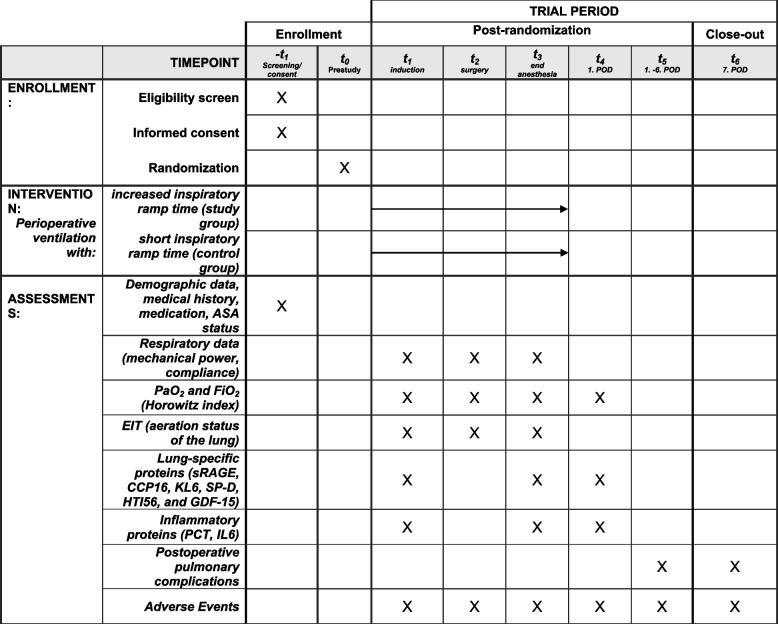
Timeline

The participant timeline is presented in Fig. 1. On the day before surgery, eligible patients will be informed about the study by the study director or his deputy. If the patient agrees to participate in the study, age, height, sex, weight, and American Society of Anesthesiologists physical status will be recorded. On the day of surgery, a peripheral venous cannula will be placed, and standard anesthesia monitoring will be established. An EIT belt will be placed around the chest. After induction of anesthesia according to the local standard clinical protocol, the airway will be managed with a suitable endotracheal tube. After intubation, an arterial access point will be established, and the first blood sample will be collected to analyze lung-specific and inflammatory proteins. Blood gas analyses will be performed hourly, and the inspiratory oxygen fraction will be recorded. EIT measurements will be performed hourly for 5 to 10 min during mechanical ventilation. At the end of surgery, another blood sample will be taken for protein analysis. Patients will be extubated according to standard procedures and transferred to the neurosurgery intensive care unit or to the ward after a stay at the postoperative care unit. A final blood sample will be collected on the first postoperative day for protein analysis.

### Sample size {14}

Unpublished preliminary tests in a physical pulmonary model showed that an increased inspiratory ramp time could reduce mechanical power compared to a short inspiratory ramp time with a partial Eta^2^ of 0.25. Using an ANCOVA with predicted body weight, mean ventilation volume and surgery time, 13 patients per group are needed for gaining a power of 0.8 at a level of significance of 0.05. With an expected dropout rate of 8%, a total of 28 patients are required.

### Recruitment {15}

The patients will be recruited based on named specific inclusion and exclusion criteria. All patients scheduled for elective surgery at the Department of Neurosurgery will be screened by the study personnel. Those who meet the inclusion criteria will be informed about the study and receive informational material by the study director or his deputy. Recruitment is independent of gender, origin, or social status.

## Assignment of interventions: allocation

### Sequence generation {16a}

Participants will be randomly assigned to one of two groups: Ventilation with an increased inspiratory ramp time (study group) or ventilation with a short inspiratory ramp time (control group). Randomization will be done in a 1:1 ratio. The assignment will not depend on any patient factors. A computer-generated block randomization with blocks containing 2 to 6 patients will be used to create a random grouping sequence. The study team will have no access to the randomization list.

### Concealment mechanism {16b}

The allocation will be done using the closed envelopes technique.

### Implementation {16c}

The envelope will be opened by the data collector right before anesthesia starts. The data collector will inform the anesthesiologist about the ventilation settings and will hand out the corresponding ventilation protocol.

## Assignment of interventions: blinding

### Who will be blinded {17a}

During the intervention period, only the patients will be blinded to group allocation. The attending anesthesiologist will necessarily be unblinded during mechanical ventilation but will not be involved in data collection or analysis. Blinding of the data collector will not be feasible in the perioperative period due to his task to ensure adherence to the ventilation protocol. The data collector will take the blood samples and record the ventilation data and the EIT data. The assessor evaluating postoperative pulmonary complications will be blinded to group assignment. All recorded data and all blood samples will be labeled with pseudonyms that do not reveal group assignment, ensuring laboratory personnel and outcome assessors will be blinded. Only during the final overall evaluation can the connection to the group membership be traced back.

### Procedure for unblinding if needed {17b}

Unblinding of the patients upon request is possible after the period of data collection, i.e., after the seventh postoperative day.

## Data collection and management

### Plans for assessment and collection of outcomes {18a}

To measure mechanical power, flow rate and pressure curves will be continuously recorded during the whole period of mechanical ventilation. The data will be further processed with Matlab (R2024a, The MathWorks Inc., Natick, USA). For each breath, mechanical power will be calculated as the area circumscribed by the inspiratory part of the volume-pressure loop and the y-axis after subtracting the resistance of the endotracheal tube. The resulting values will be averaged over the entire ventilation period. These mean mechanical power values will then be compared between the two groups.

In addition, further variables of the respiratory system mechanics like mean airway pressure or compliance will be calculated for each breath. To quantify how much compliance changes for each patient during mechanical ventilation, the average compliance after intubation will be set as “baseline compliance.” This baseline value will be compared to the average compliance of the following period of mechanical ventilation. Mean airway pressure and changes in compliance will be compared between the groups.

Continuous impedance measurement will start during induction of anesthesia until 10 min after intubation (EIT induction). Subsequently, samples of a 5- to 10-min period will be taken every hour for each patient. Data will be recorded at a sampling frequency of 50 Hertz and converted into binary data using EIT Data Analysis Tool 6.1 and further evaluated with Matlab. Mean impedance will be calculated by averaging all resistance values over each 5- to 10-min period and will be shown descriptively over time. In addition, the center and distribution of ventilation will be analyzed.

Furthermore, blood gas analyses will be performed every hour from the start of ventilation to measure arterial oxygen partial pressure. From this, the Horowitz index will be calculated as the quotient of blood carbon dioxide partial pressure and inspiratory oxygen fraction. The Horovitz indices for patients will be described over time and compared between groups.

Blood samples will be collected after induction of anesthesia, at the end of surgery, and on the first postoperative day to measure the concentration of six lung-specific proteins: soluble receptor for advanced glycation end products (sRAGE), clara cell protein 16 (CCP16), Krebs von den Lungen-6 (KL6), surfactant protein D (SP-D), human type I cell-specific apical membrane protein (HTI56), and growth differentiation factor 15 (GDF-15). The concentrations of the six biomarkers will independently be measured in the laboratory using an Enzyme-Linked Immunosorbent Assay (ELISA). After collection, blood samples will be centrifuged at 2000 × g for 15 min, and the serum will be stored at −80 °C until analysis. For each patient, protein concentrations of all three collection times will be determined and documented. The concentration of the first sample will be used as the baseline. The following concentrations will be compared to this baseline (fold-change). Changes in lung-specific protein levels over time and differences between control and intervention groups will be described.

Additionally, concentrations of two inflammatory biomarkers (PCT and IL-6) will be determined by the Institute of Clinical Chemistry and Laboratory Medicine of the Medical Center. Both the progression of concentrations and the difference between the groups will be analyzed.

Postoperative pulmonary complications will be evaluated for the following 7 days after surgery according to a standardized protocol. For this purpose, the entire patient record and all available results of medical examinations will be screened. Pulmonary complications include the following: Mild to severe respiratory failure, necessity of additional oxygen demand, reintubation or noninvasive ventilation therapy, occurrence of pneumonia, pulmonary edema, pneumothorax, pleural effusion, atelectasis, bronchospasm, or acute respiratory distress syndrome. The rates of these complications will be compared between the two groups.

### Plans to promote participant retention and complete follow-up {18b}

The study period for the primary outcome is completed after surgery. For further secondary endpoints, patient records will be reviewed. A formal participant retention plan is not needed.

### Data management {19}

The data sources include the anesthesia protocol, measured variables (such as ventilation parameters, Horovitz-index and EIT data), and measured protein concentrations. Patient records are also part of the data sources. The data collected in this study are recorded and processed digitally. Patient and disease information is handled confidentially, following data protection laws. These data are stored in a pseudonymized form. Patient data and study data are kept separate from each other. Allocation lists and signed consent forms are stored in different folders on site. Only medical and scientific staff involved in the study can access it.

### Confidentiality {27}

The medical and scientific staff in this clinical study follow medical confidentiality rules and data protection laws to ensure confidentiality of the data. Digital data will be stored securely at the study site and non-digital written data and participant information will be stored in areas with restricted access. All data will be pseudonymized to prevent patient identification, and the pseudonym list, where pseudonym and identity are linked, will be stored separately with access limited to the study director. After complete evaluation of the data, the pseudonym list will be destroyed so that the data become fully anonymized. Any published results will protect the identities of the patients involved.

### Plans for collection, laboratory evaluation and storage of biological specimens for genetic or molecular analysis in this trial/future use {33}

Blood samples will be collected for analysis of lung-specific and inflammatory proteins. Inflammatory proteins will be measured by the colleagues of the Institute of Clinical Chemistry and Laboratory Medicine. Blood samples will be destroyed immediately after testing. Blood samples for analysis of the lung-specific proteins will be stored in the working group laboratory underlying restricted access, until all patient samples have been collected and analyzed by ELISA. After quality and validity check of the results, the remaining blood samples will be destroyed. Any study-related data, including consent forms, will be deleted 10 years after the first publication.

## Statistical methods

### Statistical methods for primary and secondary outcomes {20a}

All results will be analyzed on an as-treated basis. Statistical analysis will be performed using SPSS 29.0.0 software (SPSS Statistics for Windows, IBM, Armonk, NY, USA). Variables will be expressed as mean ± standard deviation or with a 95% confidence interval as appropriate. All statistical tests will be performed with bilateral tests and a *p*-value < 0.05 will be valued as statistically significant.

1. Statistical methods for primary outcome
  The study aims to compare the average mechanical power during the ventilation period between the two groups. This will be done with a two-sided ANCOVA. Predicted body weight, mean minute volume, and surgery time will be used as covariates.
2. Statistical methods for secondary outcomes
  A linear mixed model with duration of mechanical ventilation and group membership as fixed effects will be used to compare the Horovitz index between the two groups over time. A linear regression with a two-sided 95% confidence interval will be used to estimate the effect of a long versus a short inspiratory ramp time on mean airway pressure and ΔC. The model also includes duration of mechanical ventilation and baseline compliance. Changes in global electrical impedance of the lungs, area, and center of ventilation will be analyzed descriptively. The changes of lung-specific protein concentrations based on sampling times within each group will be analyzed with a linear mixed model. A t-test will be used to compare changes in these protein levels at different time points between the two groups. The change of concentrations of the inflammatory proteins will be described. Postoperative pulmonary complications will be analyzed descriptively.

### Interim analyses {21b}

One major interim analysis is planned after complete data collection of the 12th patient. A complete review of complications during mechanical ventilation and postoperative complications will be done with a special focus on intervention-associated complications. If any identified complications could be associated with this investigation, recruitment will be temporarily suspended until further actions are discussed with the local ethics committee. During the whole investigation postoperative pulmonary events will be monitored. All patients will be screened electronically 7 days after joining the study. If there are any study-related complications identified, this will be reported immediately to the study director. Further actions will then be taken as needed. If serious adverse events will be detected during the investigation, the study director will quickly inform the Ethics Committee. Patient recruitment will stop until the issues are clarified.

### Methods for additional analyses (e.g., subgroup analyses) {20b}

There are no additional analyses planned.

### Methods in analysis to handle protocol non-adherence and any statistical methods to handle missing data {20c}

If data for the primary outcome is missing at the conclusion, exceeding the number of the expected drop-out rate, additional patients will be recruited after agreement of the local Ethics Committee. Missing data of other variables will not be supplemented.

### Plans to give access to the full protocol, participant level-data and statistical code {31c}

Due to the sensitivity of patient data, access to full protocol, participant level-data and statistical codes will be provided in anonymized form after reasonable request. The complete study protocol will be shared on reasonable request.

## Oversight and monitoring

### Composition of the coordinating centre and trial steering committee {5d}

The project was planned by Dr. med. Johannes Hell and his deputy, Prof. Dr. rer. nat. Stefan Schumann. They are both responsible for conducting the study and analyzing the data. Jonas Manke, a doctoral candidate, will ensure protocol adherence, ensure monitor data recording, and support the measurement of the lung-specific protein levels with ELISAs.

### Composition of the data monitoring committee, its role and reporting structure {21a}

In accordance with the local Ethics Committee a Data Monitoring Committee is not necessary for this study due to its low risk for participants.

### Adverse event reporting and harms {22}

Adverse events will be reported to the study leader and evaluated particularly with regard to study-related measures. Serious events will be reported to ethics committees if required, and documented in the study files for review.

### Frequency and plans for auditing trial conduct {23}

An official auditing is not required for this trial.

### Plans for communicating important protocol amendments to relevant parties (e.g., trial participants, ethical committees) {25}

Important protocol changes will be announced to the local Ethics Committee as an amendment by the director of the study. All changes will be recorded.

### Dissemination plans {31a}

The results of the study will be shared at scientific conferences and published in scientific journals. The rules for Good scientific practice and Good scientific practice authorship will be respected.

## Discussion

Despite recent advances, mechanical ventilation is still an unphysiological transfer of mechanical energy from the ventilator to patient’s lungs, which necessitates further improvement. Furthermore, detecting and discriminating even small improvements in mechanical ventilation is challenging, because so far, no sensitive biological parameters were identified, which reliably reflect mechanical load on the respiratory system and its consecutive biological trauma. This study aims to address this gap by evaluating pressure-controlled ventilation with volume guarantee using a reduced inspiratory flow rate. The reduction will be achieved by increasing the inspiratory ramp time, a simple modification, for which unpublished preliminary data from a physical model suggests that it may reduce peak wall shear stress in the airways, smoothen alveolar stretch, and decrease the mechanical load transferred to the lungs. Even though many studies on lung-protective ventilation mention the reduction of flow rate as a potential target [1–8], only limited data of clinical trials exist. One reasonable cause might be that reduced inspiratory flow affects the inspiration, reflecting only one third to one half of the whole breathing cycle. Thus, its overall impact on lung-protective ventilation may have only minor contribution to lung-protective ventilation. However, because reducing inspiratory flow rate is very easy to accomplish without any modifications of the ventilator by increasing the inspiratory ramp time during pressure-controlled ventilation, we consider it a promising target, even if only small effects are expected. To quantify the mechanical consequences of this modification, mechanical power will be used as the primary measure of mechanical load transferred from the respirator to the respiratory system. Mechanical power seems to be the most suitable value to detect and discriminate even small changes in transferred mechanical load [1, 3]. In addition, we will investigate several lung-specific proteins in patient’s blood to improve the biological assessment of mechanical load and potential ventilator-associated injury.The analyses of the kinetics of these lung-specific proteins and their concentration in combination with the results of the calculated mechanical energy transferred to the lungs might facilitate detecting a more suitable biological parameter, representing mechanical load of the lungs. The selected markers include sRAGE [15, 16] and GDF-15 [17], which are among the most extensively studied but not fully lung-specific proteins; HTI-56 as a marker of typ-I pneumocytes [18, 19], KL-6 and SP-D as markers of type-II pneumocytes [20] and CCP-16, representing the bronchioles [21]. This selection of proteins may also help to identify the anatomical region of pulmonary injury. However, pulmonary damage is subjected to a complex and multifactorial pathogenesis, where even a non-pulmonary inflammatory reaction could promote lung damage [22, 23]. Therefore, we investigated patients receiving neurosurgery to obtain a long time of mechanical ventilation with limited surgical tissue trauma. Furthermore, to control inflammatory response, IL 6 and PCT will be measured to quantify systemic inflammatory reaction.

Beyond biological markers and mechanical power, our study will evaluate respiratory system mechanics, Horowitz index, distribution of ventilation, and postoperative pulmonary complications, to cover a broad range of relevant outcomes and potential differences between the two ventilation strategies.

We are aware of several limitations. First, the number of patients is small. However, this first trial focuses on perioperative feasibility and the safety of ventilation modification while conserving resources and minimizing patient risks. If the study results suggest clinically relevant benefits, an extended multicenter investigation will be planned. Second, blinding of the data collector and the attending anesthetist is not possible, which may influence perioperative management or data completeness. Nevertheless, mechanical ventilation will be strictly implemented according to the study protocol. Data recording will be independent, and all analyses will be performed under blinded conditions. Third, besides inspiratory ramp time, the two ventilation strategies differ in the inspiratory-to-expiratory time ratio. Therefore, differences in the mechanical load cannot be attributed solely to the inspiratory ramp time. However, we deliberately chose a reduced inspiratory-to-expiratory time ratio in the study group to facilitate the longest possible inspiratory ramp time to accentuate differences. We are aware that this point must be taken into account in any conclusion. Fourth, the selection of lung-specific proteins is limited. However, additional markers will be analyzed if the planned analysis is inconclusive.

Besides inspiratory ramp time, the two ventilation strategies differ in the inspiratory-to-expiratory time ratio. Therefore, differences in the mechanical load cannot be attributed solely to the inspiratory ramp time. However, we deliberately chose a reduced inspiratory-to-expiratory time ratio in the study group to facilitate the longest possible inspiratory ramp time to accentuate differences. We are aware that this point must be taken into account in any conclusion.

In conclusion, this trial investigates a simple modification aimed at reducing inspiratory peak flow as another potential component of lung-protective ventilation. By combining mechanical measurements with detailed biological profiling, the study aims to improve the assessment and discrimination of lung-protective ventilation. This research could therefore lead to a new easily applicable form of lung-protective ventilation. Identifying a lung-specific protein, which represents mechanical load of the lungs could improve risk-stratification and diagnosis or therapy of patients with lung damage.

### Trial status

Protocol version 2.0 was approved by the local ethics committee on 17 September 2024 and version 3.0 including an amendment was approved on 14 August 2025. Patient recruitment began on 2nd September 2025. At the time of writing the manuscript, one-third of patients were successfully enrolled. Recruitment is expected to be completed by February 2026.

## Data Availability

The datasets used and/or analyzed during the present study are available from the corresponding author on reasonable request.

## Abbreviations

CCP16: Clara cell protein 16
EIT: Electrical impedance tomography
ELISA: Enzyme-Linked Immunosorbent Assay
GDF-15: Growth differentiation factor 15
IL-6: Interleukin 6
HTI56: Human type I cell-specific apical membrane protein
KL6: Cancer of the lungs-6
PCT: Procalcitonin
SP-D: Surfactant protein D
sRAGE: Soluble receptor for advanced glycation end products
